# Systemic Dosing of Thymosin Beta 4 before and after Ischemia Does Not Attenuate Global Myocardial Ischemia-Reperfusion Injury in Pigs

**DOI:** 10.3389/fphar.2016.00115

**Published:** 2016-05-03

**Authors:** Christoffer K.-J. Stark, Miikka Tarkia, Rasmus Kentala, Markus Malmberg, Tommi Vähäsilta, Matti Savo, Ville-Veikko Hynninen, Mikko Helenius, Saku Ruohonen, Juho Jalkanen, Pekka Taimen, Tero-Pekka Alastalo, Antti Saraste, Juhani Knuuti, Timo Savunen, Juha Koskenvuo

**Affiliations:** ^1^Research Center of Applied and Preventive Cardiovascular Medicine, University of TurkuTurku, Finland; ^2^Heart Center, Turku University Hospital and University of TurkuTurku, Finland; ^3^Turku PET Centre, Turku University Hospital and University of TurkuTurku, Finland; ^4^Department of Anesthesiology, Intensive Care, Emergency Care and Pain Medicine, Turku University HospitalTurku, Finland; ^5^Children’s Hospital, Pediatric Cardiology, Helsinki University HospitalHelsinki, Finland; ^6^Department of Vascular Surgery, Turku University Hospital and University of TurkuTurku, Finland; ^7^Department of Pathology, Turku University Hospital and University of TurkuTurku, Finland

**Keywords:** thymosin beta 4, cardiopulmonary bypass, ischemia-reperfusion injury, cardioprotection, coagulation, vasoconstriction

## Abstract

The use of cardiopulmonary bypass (CPB) and aortic cross-clamping causes myocardial ischemia-reperfusion injury (I-RI) and can lead to reduced postoperative cardiac function. We investigated whether this injury could be attenuated by thymosin beta 4 (TB4), a peptide which has showed cardioprotective effects. Pigs received either TB4 or vehicle and underwent CPB and aortic cross-clamping for 60 min with cold intermittent blood-cardioplegia and were then followed for 30 h. Myocardial function and blood flow was studied by cardiac magnetic resonance and PET imaging. Tissue and plasma samples were analyzed to determine the amount of cardiomyocyte necrosis and apoptosis as well as pharmacokinetics of the peptide. *In vitro* studies were performed to assess its influence on blood coagulation and vasomotor tone. Serum levels of the peptide were increased after administration compared to control samples. TB4 did not decrease the amount of cell death. Cardiac function and global myocardial blood flow was similar between the study groups. At high doses a vasoconstrictor effect on mesentery arteries and a vasodilator effect on coronary arteries was observed and blood clot firmness was reduced when tested in the presence of an antiplatelet agent. Despite promising results in previous trials the cardioprotective effect of TB4 was not demonstrated in this model for global myocardial I-RI.

## Introduction

Cardiopulmonary bypass (CPB) assisted open-heart surgery allows the surgeon to work in a quiet and bloodless operating field and is mandatory in many routine types of cardiac surgery. During CPB the heart is stopped and isolated from the blood circulation while protected from ischemia with cardioplegic solution. After ischemia, the return of myocardial blood flow (MBF) leads to reperfusion injury causing further damage to the heart. Ischemia-reperfusion injury (I-RI) can be identified by biomarkers for cardiomyocyte death, reduced cardiac function and the incidence of arrhythmias (Anselmi et al., 2004; Nesher et al., 2008; Hausenloy et al., 2012). Disrupted microvascular blood flow is usually seen after revascularization procedures but is also present after protected I-RI (Boyle et al., 1996; Verrier and Morgan, 1998; Wong et al., 2013). Extracorporeal circulation causes a systemic inflammatory response that can lead to organ dysfunction and hemodynamic instability (Hausenloy et al., 2013). Different approaches to minimize the effects of myocardial I-RI have been studied extensively, there is, however, little agreement regarding the most optimal cardioprotective strategy in heart surgery. Especially high-risk patients could benefit from more effective methods attenuating I-RI (Bolli et al., 2004).

Thymosin beta 4 (TB4) is a naturally occurring g-actin sequestering peptide that is expressed in most cell-types and is especially abundant in inflammatory cells and thrombocytes. Some reports have showed an important role for the peptide during cardiovascular organ development although there is some debate regarding this argument. In adulthood the effects are thought to be related to tissue homeostasis and repair (Smart et al., 2010; Banerjee et al., 2012; Goldstein et al., 2012; Rossdeutsch et al., 2012). At sites of tissue injury the peptide is released in high quantities and bound to fibrin and fibrinogen potentially increasing its bio-availability (Goldstein et al., 2012). TB4 has previously proved its remarkable therapeutic potential in several studies using rodent models for myocardial injury and is thought to aid in both protecting and repairing the heart. During global myocardial I-RI the peptides anti-inflammatory and cell-survival promoting properties could be valuable (Bock-Marquette et al., 2004; Sosne et al., 2010; Stark et al., 2012; Bollini et al., 2015).

We wanted to test the cardioprotective effect of TB4 in a clinically relevant surgical model for cardiac I-RI in the subacute phase and also investigate some of its effects on tissue responses related to injury. Pigs underwent CPB and cardioplegic heart arrest for 60 min and were followed for 30 h. TB4 or vehicle was administered before ischemia and after reperfusion of the heart. The cardioprotective effects of peptide were analyzed by cardiac magnetic resonance (CMR) and ^15^O-water positron emission tomography (PET) imaging as they are well validated in assessing myocardial function, viability and blood flow (Viswamitra et al., 2004; Iida et al., 2012). Biochemical and histological analyses were performed for pharmacokinetic studies of TB4 and to determine the amount of cardiomyocyte apoptosis and necrosis. The knowledge of the peptides’ effects on blood clotting and arterial contraction is of importance in any surgical model with regard to risk for bleeding and thrombosis but also when considering TB4 treatment in thromboembolic conditions such as myocardial infarction or when planning direct intracoronary delivery. Therefore we also investigated the peptides influence on vasomotor tone and on blood coagulation *in vitro*.

## Materials and Methods

### Anesthesia, Surgery, and Monitoring

Ten landrace pigs weighing 29–43 kg were operated on and divided into a TB4 treatment group (*n* = 6) and a control group (*n* = 4). All animal experiments were approved by the Laboratory Animal Care & Use Committee of the State Provincial Office of Southern Finland.

The pigs were pre-anesthetized with an intramuscular injection of 100 mg xylazin (Rompun vet, Bayer Animal Health GmbH, Leverkusen, Germany) and 25 mg midazolam (Midazolam Hameln, Hameln pharmaceuticals GmbH, Hameln, Germany). A peripheral ear vein was cannulated and the animals were medicated with 20 mg boluses of propofol (PropofolLipuro, B. Braun Melsungen AG, Melsungen, Germany) and 150 μg phentanyl (Fentanyl-Hameln, Hameln pharmaceuticals GmbH, Hameln, Germany) and were then intubated and connected to a respirator (Dräger Oxylog 3000, Drägerwerk AG, Lübeck, Germany). The respiratory rate was set to 18–22 times/min with a tidal volume of 8–10 ml/kg using 40% oxygen. Blood gases were monitored to ensure adequate ventilation (i-STAT, Abbott Laboratories, Abbott Park, IL, USA). Anesthesia was maintained with a continuous infusion of propofol 15–30 mg/kg/h, phentanyl 1.5 μg/kg/h and midazolam 100 μg/kg/h. Preoperatively and every 8 h thereafter the animals received antibiotic prophylaxis (Cefuroxime 750 mg, Orion Pharma, Espoo, Finland). Before the heart was cannulated 10000 IU of heparin (Heparin, LEO Pharma, Ballerup, Denmark) was given as a bolus and the dose was repeated every 30 min during extracorporeal circulation. Finally the heparin was neutralized by administering 14000 IU of protamine sulphate (Protamin, LEO Pharma, Ballerup, Denmark). TB4 (supplied by RegeneRx Biopharmaceuticals Inc, Rockville, MD, USA) was suspended in sterile water to a concentration of 4 mg/ml (0.82 mM) and administered 6 mg/kg through a 15 min intravenous infusion 30 min before the start of the operation and 6 h later.

The right external jugular vein was cannulated for invasive central venous pressure monitoring and drug administration. The femoral artery was cannulated for blood pressure monitoring and blood sampling. Invasive hemodynamic parameters, ECG and oxygen saturation were monitored throughout the study (DatexOhmeda S5, GE Healthcare Finland Oy, Helsinki, Finland). A percutaneous cystostomy was performed for urine output monitoring.

Since pigs lack blood groups, a blood donor littermate was used to prime the perfusion lines of the heart–lung machine. The donors underwent the same anesthetic protocol and their blood was drained after heparin administration from the right external jugular vein via a central venous catheter into the reservoir of the heart–lung machine.

The pigs were placed in a left lateral decubitus position and the chest was shaved and disinfected. The animals were draped and a thoracotomy was performed in the fourth intercostal space on the right. A rib retractor was used to gain access to the heart. The lung was displaced and the pericardium opened. Purse string sutures were placed on the ascending aorta and on the right auricle for cannulation. The aorta was cannulated with a pediatric 14 F cannula and the right auricle with a sequential 24/28 F cannula (Medtronic, Minneapolis, MN, USA). A 5 F cannula was placed at the root of the aorta for cardioplegia infusion and venting of the heart. Custom-made perfusion lines were connected between the animal and a pediatric membrane oxygenator (Dideco 905 Eos, Dideco, Mirandola, Italy) and perfusion was initiated. The aorta was cross-clamped and 500 ml of cold (10°C) Modified St Thomas Hospital No II cardioplegia was administered with blood in a 4:1 ratio via the ascending aorta. A second dose of cardioplegia was given after 30 min. During perfusion the animals were kept normothermic (36–38°C) and the mean arterial pressure was kept at 50–60 mm Hg. Blood lactate levels were monitored to ensure adequate perfusion. After 60 min the aortic cross-clamp was removed and the heart was defibrillated as necessary. Some animals required re-clamping of the aorta with a short cardioplegic arrest due to persistent ventricular fibrillation. For rhythm disorders 100–150 mg of lidocaine (Lidocain, Orion Pharmaceuticals, Espoo, Finland) and 150–225 mg of amiodarone (Cordarone, Sanofi, Helsinki, Finland) was administered as needed. The heart was vented and the animals were allowed to wean from CPB until the heart had recovered and was generating pulsatile flow. Boluses (5 mg) of ephedrine (Efedrin, Stragen Nordic, Hilleroed, Denmark) and infusion (80–160 μg/h) of norepinephrine (Noradrenalin Hospira, Hospira UK Limited, Warwickshire, UK) was used as needed for postoperative hemodynamic support. After weaning, the animals were decannulated. A chest tube was left in the right pleural cavity and connected to negative pressure. The thoracotomy wound was closed in three layers. The wound was infiltrated with 50 mg of bupivacaine (Bicain, Orion Pharmaceuticals, Espoo, Finland) for post-operative analgesia. 20 mg of enoxaparin (Klexane, Sanofi, Helsinki, Finland) was given for thrombosis prophylaxis 1 and 12 h after the operation. The animals were kept on mechanical ventilation for the complete duration of the experiment. Hemodynamic parameters were continuously monitored and regular blood samples were drawn for hemoglobin, blood glucose, and pH measurements. Cardiac troponin T (cTnT) release was measured from plasma samples at baseline, 6 and 24 h post-reperfusion by electro-chemiluminescence immunoassay (Elecsys^®^Troponin T high sensitive, Roche Diagnostics Ltd.) performed by the Hospital District of Southwest Finland laboratory services. The animals were finally sacrificed by an intravenous injection of potassium chloride.

### Thymosin Beta 4 EIA

Serial serum samples were collected from two animals in both groups for pharmacokinetic studies of TB4 by EIA. The serum was prepared and analyzed as parallel samples according to the manufacturers’ manual (Thymosin β4 EIA Kit, Immundiagnostik AG, Bensheim, Germany). A coefficient of variation (calculated as: sample SD/sample mean) of less than 50% between parallel samples was accepted for final analysis.

### Artery Wire Myography

Eighteen representative segments of small mesentery arteries and coronary arteries (250–350 μm in diameter) from 4 blood-donor pigs were studied by wire myography for contractile responses to TB4 *in vitro*. Samples were excised rapidly and placed in ice-cold oxygenated Krebs solution. The composition of the Krebs solution was as follows (in mM): 119 NaCl, 4.7 KCl, 25 NaHCO3, 1.2 NaH2PO4, 2.5 CaCl2, 1.2 MgSO4, and 5.5 glucose. Arterial rings (2 mm in length) were mounted using 40 μm wires in a microvessel myograph (Danish Myograph Technologies, Aarhus, Denmark) for isometric tension recordings. After mounting, vessels were equilibrated, normalized and contracted repeatedly with 62 mM potassium chloride until maximal and reproducible contractions were obtained. To examine the contracting effects of TB4, dose-response curves to TB4 (10-8-10-2 M) were constructed and compared to KCl-induced contraction. Relaxation was studied in thromboxane (TXA2) precontracted arterial rings. The precontraction was adjusted to 20–40% of the reference contraction to 62 mM KCl, which after cumulative concentrations of TB4 was added to the organ bath. To evaluate the role of NO on the effects of TB4 induced vasodilation, experiments were repeated in arteries incubated with L-NNA, a NO synthase inhibitor (100 μM, 30 min). Data were collected and analyzed using Powerlab and Chart5 softwares (ADI Instruments, Colorado Springs, CO, USA).

### Thromboelastometry Analysis

Rotem^®^thromboelastometry (Tem Innovations GmbH, Basel, Switzerland) analysis was performed *in vitro* on blood samples from three blood-donor animals. TB4 was added to citrated whole blood to achieve two different concentrations (0.1 mM and 1 mM) and incubated at room temperature for 1 h. Untreated blood samples served as controls. The samples were analyzed and three parameters were drawn from the results at 60 min: Maximum clot firmness (stability of the blood clot), clotting time (time to clot formation), and maximum lysis of the blood clot. Assays used were EXTEM, INTEM, and FIBTEM. EXTEM measures the activity of the extrinsic pathway with tissue factor activation and INTEM of the intrinsic pathway with contact activation. FIBTEM is used for the assessment of fibrinogen status by complete platelet inhibition.

### Tissue Sample Harvesting and Apoptosis Detection

Transmural tissue samples from the left and right ventricles were collected and fixed in formalin. Hematoxylin and eosin staining was performed for general histological analysis. Cardiomyocyte apoptosis was determined by terminal transferase nick end-labeling assay (TUNEL) as previously described (Gavrieli et al., 1992; Saraste et al., 1997). Positive apoptotic nuclei were counted over an ocular grid and presented as a percentage of total cardiomyocyte number in 10 randomly selected microscopic visual high power (×40) fields by an investigator blinded to group assignments.

### Cardiac Positron Emission Tomography

The pigs underwent cardiac PET scans 25–26 h post-reperfusion to determine MBF during rest and adenosine stress. PET studies were performed with ECAT EXACT HR+ scanner (Siemens-CTI, Knoxville, TN, USA). Myocardial perfusion was evaluated by PET with ^15^O-radiolabeled water (808 ± 129 MBq) both at rest and during intravenous adenosine stress (200 μg/kg/min). The acquisition frames were as follows: 14 × 5 s, 3 × 10 s, 3 × 20 s, and 4 × 30 s (total duration 4 min 40 s). The acquired PET data were reconstructed in 2D mode with an iterative reconstruction algorithm OSEM using six iteration and 16 subsets in the reconstruction. The transaxial field of view (35 cm) was reconstructed in a 128 × 128 matrix, yielding a pixel size of 2.57 mm × 2.57 mm. The measurements were corrected for scatter, random counts, and dead time. The device produces 63 axial planes with a slice thickness of 2.43 mm. Global myocardial perfusion (ml/g/min) was measured using Carimas 2 software (Turku PET Centre, Turku, Finland^1^).

### Cardiac Magnetic Resonance Imaging

Four animals in each group underwent cardiac magnetic resonance imaging (MRI) 27–30 h post-reperfusion. MRI study was performed at 1.5 T system (Philips GyroscanIntera Nova Dual MR, Philips Medical Systems, Best, The Netherlands) with a phased-array torso coil and a vector cardiographic method for ECG-gating. All acquisitions were obtained during short breath hold periods. Each MRI study consisted of cine imaging of both ventricles for volumetrics and wall motion at rest. Late enhancement (LE) imaging was applied for scar assessment. MRI analysis was performed as showed in our previous studies and an experienced reader, blinded to therapy, analyzed all MRI exams (Koskenvuo et al., 2007; Mäki et al., 2012). bTFE pulse sequences were used with the following parameters: retrospective gating, repetition time/echo time (TR/TE) 3.4/1.2 ms, flip angle 60, FOV 320–360 mm, acquisition matrix 192 × 256, reconstruction matrix 256 × 256, rFOV 100%, 30 phases/cardiac cycle, slice thickness 6.0 mm, and gap 0 mm. A phase-sensitive inversion recovery T1–TFE pulse sequence was applied for LE imaging 10 min after injecting gadoterate meglumine (Dotarem 279.3 mg/mL, Guerbet, Roissy CdG, France) with a dose 0.3 mmol/kg. The following imaging parameters were used: prospective gating, TR/TE 4.0/1.2 ms, flip angle 15°, acquisition matrix 168 × 256, reconstruction matrix 256 × 256, FOV 320–360 mm, rFOV 100%, 8–15 slices/breath hold, slice thickness 6 mm, and gap 0 mm. TI was optimized individually using a TI-scout sequence. The whole LV was covered by LE images at SA orientation.

### Statistical Analysis

Statistical power was calculated with an estimation of a 10% increase in LVEF with TB4 treatment (alpha 0.05). A power value of 0.8 gave a sample size of 4–6/group. Data is presented as mean ± SD or mean ± SEM for myography results. Paired and unpaired *t*-tests were used for single comparative analysis. Two-way ANOVA and Tukey’s multiple comparisons test was used for multiple comparative analysis. A *p*-value of less than 0.05 was considered significant in all analyses.

## Results

In the TB4 treatment group two animals did not complete the follow-up period. One pig died shortly after weaning from CPB due to bradycardia and the other one 20 h after reperfusion due to circulatory failure. Four animals in each group completed the study and underwent all planned analyses.

Preoperative and perioperative variables were similar (**Table 1**). The use of inotrope and vasopressor agents in the immediate post-operative period did not differ between the study groups. All animals received norepinephrine infusions for short periods of time and small doses of ephedrine. Two animals in each group required amiodarone and lidocaine therapy for persistent ventricular fibrillation, the dosage was, however, equal between the groups. Three animals in both groups required re-clamping of the aorta and a short cardioplegic re-arrest due to inability to terminate ventricular fibrillation. The total aortic cross-clamp times did, however, remain similar between the study groups. Blood pressure and heart rate values were consistent throughout the follow-up period. No direct effect of TB4 treatment was observed on these parameters (**Table 2**). Analyzed blood samples showed no signs of hemolysis.

**Table 1:** Perioperative variables of the operated animals (mean ± SD, range min–max).

	Control (*n* = 4)	Thymosin beta 4 (*n* = 4)	*P*-value
Weight (kg)	31.8 ± 1.9 (30.0–34.3)	34.4 ± 6.3 (29.1–43.0)	0.46
Procedure length (min)	192 ± 49 (165–265)	196 ± 31 (155–225)	0.91
Perfusion time (min)	106 ± 14 (95–125)	107 ± 5 (100–112)	0.82
Aortic cross–clamp time (min)	63.5 ± 2.9 (60.0–67.0)	61 ± 0.8 (60.0–62.0)	0.15
Priming volume (ml)	1300 ± 258 (1000–1600)	1400 ± 163 (1200–1600)	0.54
Cardioplegia (ml)	631 ± 47 (600–700)	575 ± 50 (500–600)	0.15

**Table 2:** Hemodynamics of the operated animals.

Heart rate (1/min)	Control (*n* = 4)	Thymosin beta 4 (*n* = 4)	*P*-value
Baseline	103 ± 15 (82 – 113)	95 ± 7 (86 – 101)	0.31
30 min	124 ± 22 (92 – 139)	114 ± 29 (76 – 145)	0.59
6 h	128 ± 19 (102 – 149)	127 ± 38 (82 – 174)	0.96
12 h	131 ± 19 (107 – 154)	137 ± 35 (102 – 180)	0.78
24 h	122 ± 1 (121 – 123)	129 ± 19 (112 – 151)	0.54
**MAP (mm Hg)**			
Baseline	66 ± 10 (57 – 81)	54 ± 7 (47 – 64)	0.10
CPB	56 ± 9 (51 – 69)	54 ± 6 (48 – 61)	0.69
30 min	63 ± 14 (43 – 76)	59 ± 8 (48 – 66)	0.60
6 h	66 ± 14 (54 – 86)	60 ± 10 (47 – 68)	0.47
12 h	61 ± 11 (44 – 70)	59 ± 9 (48 – 69)	0.87
24 h	61 ± 16 (40 – 80)	57 ± 11 (42 – 66)	0.71

*MAP, mean arterial pressure; CPB, cardiopulmonary bypass. Times are post-reperfusion (mean ± SD, range min–max).*

**FIGURE 1:**
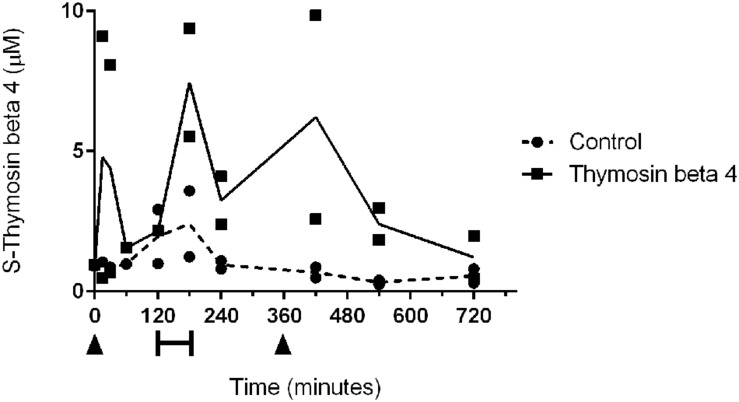
**Individual serum concentrations of thymosin beta 4 and mean concentrations in two control (dotted line) and two treated animals (black line).** Thymosin beta 4 was administered intravenously (6 mg/kg) at 0 min and at 360 min (arrowheads). Bar indicates ischemic period.

EIA analysis showed increased serum concentrations of TB4 after each dosage (**Figure 1**). The peak concentration occurred 15 min after the first dose (0.96 μM) and a second peak was observed at the start of reperfusion (1.5 μM) approximately 3 h after the first dose. An increase, although smaller than in the treated animals, of endogenous TB4 serum concentration could be seen in control animals immediately after reperfusion (0.49 μM). One hour after the second dose the serum concentration of TB4 was again elevated (1.25 μM), while it remained low in control samples.

**FIGURE 2:**
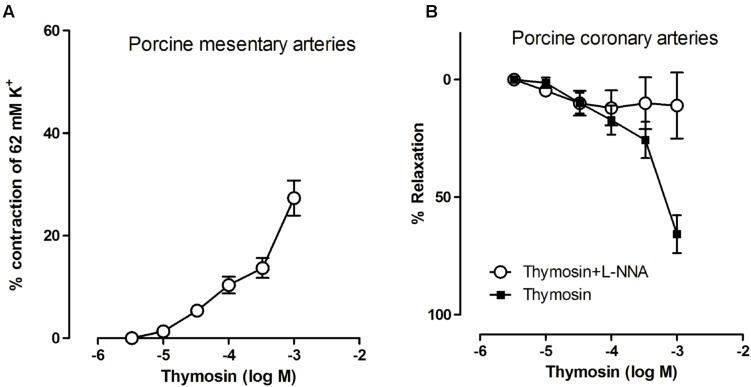
**The contractile effects of TB4 on small mesentery arteries (A) and the effects of TB4 on coronary relaxation with TB4 alone and with nitric oxide synthase inhibitor, L-NNA (B) on arteries from four animals (mean ± SEM)**.

In artery wire myography TB4 showed concentration-responsive vasoconstriction of mesenteric arteries. At concentrations of 0.1–1 mM the contractile response was 15–30% of that of maximum potassium chloride stimulation (**Figure 2A**). Similarly, TB4 showed modest endothelium-dependent relaxation in the porcine coronary arteries. The relaxing effect was abolished by a nitric oxide synthase inhibitor (L-NNA; **Figure 2B**). TB4 did not, however, induce vasodilatation in the mesenteric arteries, nor did it induce vasoconstriction in the coronary arteries.

**FIGURE 3:**
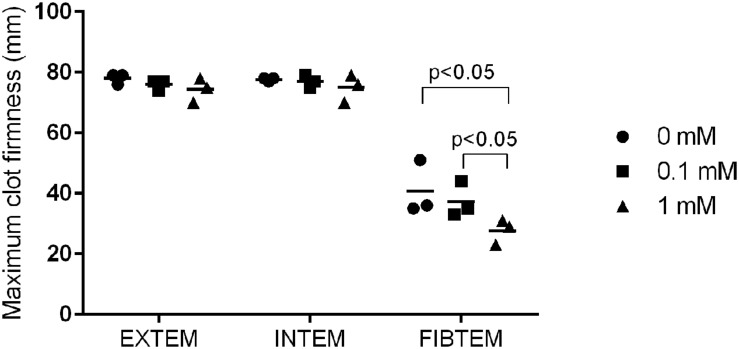
**Maximum blood clot firmness in blood samples from three animals treated with different concentrations of TB4.** EXTEM measures activity of the extrinsic and INTEM of the intrinsic pathway. FIBTEM is used for the assessment of fibrinogen status in the absence of platelets (individual values + mean).

The addition of TB4 to whole blood did not have an effect on blood clotting time or maximal blood clot fibrinolysis at concentrations used here. The clot firmness was, however, significantly reduced in the FIBTEM analysis, which measures clot firmness in the absence of functioning platelets, when comparing 1–0.1 mM and 0 mM concentrations (27.7 ± 4.2 mm versus 37.3 ± 5.9 mm versus 40.6 ± 9.0 mm, *p* < 0.05; **Figure 3**).

**FIGURE 4:**
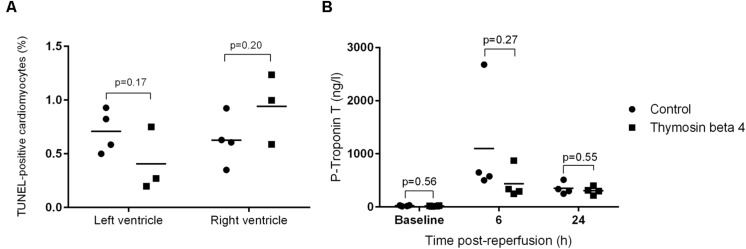
**Percentage of TUNEL-positive cells (A) in left and right ventricle tissue samples (*n* = 3 + 4) 30 h post-reperfusion and plasma concentrations of cTnT (B) 6 and 24 h post-reperfusion (*n* = 4 + 4; individual values + mean)**.

General histology showed normal tissue architecture with very little inflammatory cell infiltration. The extent of myocardial cell death did not differ between TB4 treated and control animals. TUNEL analysis showed a similar rate of apoptotic cells in both left (0.71 ± 0.2% versus 0.41 ± 0.30%, *p* = 0.17) and right (0.63 ± 0.24% versus 0.94 ± 0.33%, *p* = 0.20) ventricles (**Figure 4A**). All animals had normal baseline cTnT values (<50 ng/l). The differences in cTnT plasma concentrations at six (1100 ± 1053 ng/l versus 437 ± 291 ng/l, *p* = 0.27) and 24 h (349 ± 114 ng/l versus 305 ± 75 ng/l, *p* = 0.55) post-reperfusion did not reach statistical significance (**Figure 4B**).

**FIGURE 5:**
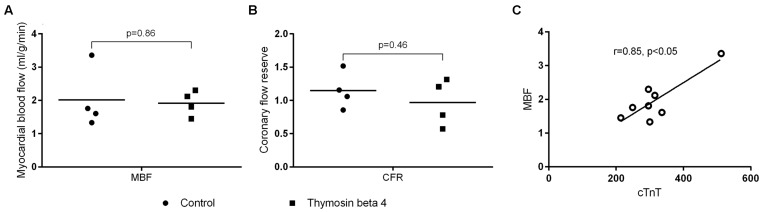
**Global myocardial blood flow (A) and coronary flow reserve (B) assessed with cardiac [^15^O]H_2_O positron emission tomography 25 h post-reperfusion (*n* = 4 + 4).** Correlation between myocardial blood flow at rest and cTnT in plasma samples **(C)** (individual values + mean).

Cardiac [^15^O]H_2_O PET studies were carried out 25 h (range 25–26) post-reperfusion to determine global MBF. Blood flow during rest was equal between control and TB4 group (2.01 ± 0.91 ml/g/min versus 1.92 ± 0.38 ml/g/min, *p* = 0.86). The values were also corrected for individual and group mean blood pressure and pulse rate products but the differences remained insignificant. Coronary flow reserve calculated from flow during adenosine stress and resting flow values were close to 1 in both groups (1.15 ± 0.28 versus 0.97 ± 0.35, *p* = 0.45). There was an association between cTnT release at 24 h and MBF at rest (*r* = 0.85, *p* < 0.05; **Figure 5**).

**FIGURE 6:**
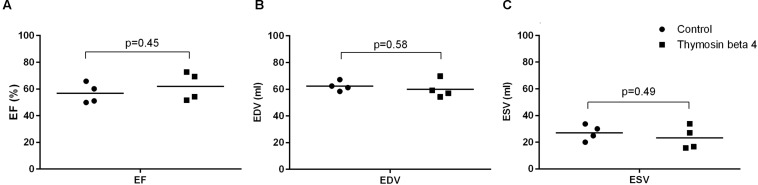
**LVEF (A), LVEDV (B), and LVESV (C) assessed by cardiac magnetic resonance imaging 28 h post-reperfusion (*n* = 4 + 4; individual values + mean)**.

Cardiac magnetic resonance imaging was performed after 28 h (range 27–30) of reperfusion. Left ventricle ejection fraction was slightly higher in the treated animals but the difference remained statistically insignificant (56.7 ± 7.6% versus 61.9 ± 10.6%, *p* = 0.45). LVEDV (62.3 ± 3.7 ml versus 60.1 ± 6.8 ml, *p* = 0.58) and LVESV (27.2 ± 6.0 ml versus 23.3 ± 8.7 ml, *p* = 0.49) did not differ between the groups (**Figure 6**). When normalized for body-surface area there was a trend for larger chamber volumes in control animals (LVEDV/BSA 40.8 ± 2.1 ml/m^2^ versus 36.7 ± 3.7 ml/m^2^, p = 0.10 and LVESV/BSA 17.7 ± 3.6 ml/m^2^ versus 14.0 ± 4.3 ml/m^2^, *p* = 0.23). One animal in the TB4 group had mild tricuspid valve regurgitation. In the other subjects there were no valvular abnormalities. No uptake of gadolinium contrast was observed on late-enhancement scans in any of the animals.

## Discussion

Thymosin beta 4 is normally found in the cytosol and also in the nucleus of cells but lacks a signal sequence for exocytosis. It is unclear whether the high concentrations found in extracellular fluids, is due to active secretion of the peptide or secondary to degranulation of inflammatory cells or cell membrane rupture (Watts et al., 1990; Huff et al., 2001). TB4 is water soluble and rapidly internalized by endothelial cells *in vitro* where it binds to actin and participates in the dynamics of the cytoskeleton (Cierniewski et al., 2012). At very high concentrations there is also some binding to f-actin, although this interaction is poorly understood (Carlier et al., 1996). Several studies have showed that intracellular TB4 increases ILK and PI3-K mediated phosphorylation of Akt which is considered responsible for many of the peptides’ cardioprotective effects (Bock-Marquette et al., 2004; Fan et al., 2009; Goldstein et al., 2012). It is reported that binding of TB4 to extracellular ATP synthase increases ATP synthesis while ATP hydrolysis is blocked. The local increase in ATP is linked to P_2X4_ mediated cell migration but signaling through other purinergic receptors is also possible (Freeman et al., 2011).

As the functions of TB4 are thought to be related to tissue homeostasis and repair after injury we wanted to test the direct vasomotor effect of the peptide in an *in vitro* setting. When using physiological concentrations of TB4 we did not observe any influence on vasoconstriction or vasodilation but when the concentration was adjusted to the 0.1–1.0 mM range, we saw an increase in vasomotor tone in mesentery arteries and a weak NO-mediated relaxation effect on coronary arteries. Pig coronaries mainly express β-adrenergic receptors while mesentery vessels express both α- and β-adrenergic receptors and this could partly explain the differences in vasomotor behavior of the two vessel types (Duncker et al., 2012). Vascular smooth muscle tone is regulated by intracellular Ca^2+^ and mediated by several cell surface receptors (Wilson et al., 2005). A previous report showed an increase in intracellular Ca^2+^ after treatment with TB4 but this was not confirmed in a more recent study (Wilson et al., 2005; Cierniewski et al., 2012). The PI3K/Akt pathway has been suggested as a regulator of calcium-dependent vasoconstriction, while pathways for Ca^2+^-independent vasoconstriction is related to ILK-mediated phosphorylation of Myosin II (Wilson et al., 2005; Huang et al., 2006; Sinagra et al., 2013). Purinergic signaling through P2 receptors is associated with vasomotor responses and is regulated either in the endothelium or by sympathetic nerve stimulation in the vessel wall (Burnstock, 2008). One possible explanation for the differences seen for coronary and mesentery artery responses could be related to ATP and a differential expression of vasodilator P_2Y_ and vasoconstrictor P_2X_ receptors within the tested arteries. We did, however, not test the responses under the influence of selective purinergic receptor antagonists. No previous data exists on TB4-mediated vasomotor activity and we can only speculate whether our findings have any clinical significance as plasma levels are normally much lower compared to concentrations tested here. Platelets, however, contain up to 0.5 mM of TB4 and at sites of blood clot formation or after pharmacological delivery of TB4, local concentrations could increase sufficiently to cause a vasomotor response mediated by known or still undiscovered targets of TB4 (Kaur et al., 2010).

It is documented that TB4 covalently binds to fibrin and fibrinogen by factor XIIIa and that his interaction inhibits platelet aggregation and adhesion at concentrations higher than 0.5 μM (Makogonenk et al., 2004; Kaur and Mutus, 2012). Based on the findings in our vasomotor experiment, we investigated whether even higher concentrations of TB4 had an influence on blood coagulation as assessed by thromboelastometry. We observed a decrease in maximal blood clot firmness with increasing concentrations from 0.1–1.0 mM when platelets were inhibited by a potent anti-platelet agent. This method is used to assess fibrinogen status in whole blood and could indicate that the fibrinogen–TB4 interaction has a negative effect on blood clot stability and might be relevant in surgical situations where the patient is also receiving anti-platelet medication. No effect on blood clotting time or fibrinolysis was observed.

The preferred dosing range of TB4 seems to be 2–12 mg/kg both when using single and repeated strategies. The most commonly used dosage of TB4 in animal studies so far is ∼6 mg/kg, as this dose has showed good therapeutic effect without signs of adverse reactions either in animal or human studies (Ruff et al., 2010; Stark et al., 2012; Morris et al., 2014). We wanted to condition the heart before the operation and also affect the immediate post-operative reperfusion period and gave the animals two doses of TB4. Under physiological conditions, concentrations of the peptide in plasma and serum is reported to be approximately 0.005–0.01 μM (Kaur et al., 2010; Ruff et al., 2010). In patients with active rheumatoid arthritis or severe coronary artery disease undergoing PCI endogenous serum levels of TB4 have been found to range from 0.1 μM up to 600 μM (Biçer et al., 2011; Song et al., 2012). In our study, the levels increased to above normal physiological levels in treated animals and remained low in controls. There is little knowledge regarding tissue pharmacokinetics of TB4 and the optimal therapeutic levels in blood or tissue. Maximal tissue concentrations in heart have been found at 2 h after intraperitoneal administration of the peptide, so we considered this time point to be suitable for the first infusion (Mora et al., 1997). In a previous study using a similar model for I-R injury, the rate of cardiomyocyte death was showed to increase already within the first hours of reperfusion (Malmberg et al., 2006). Therefore, we decided to give the second dose early during reperfusion in order to target cell-death signaling at this stage. Cardioprotection in rodents has been observed after administration both locally and systemically. We administered the peptide through intravenous infusions as this is also clinically the most feasible method (Bao et al., 2013). Tissue concentrations of TB4 were not measured, so we cannot say whether significant amounts of the peptide retained in the heart, nor did we assess whether the TB4 dose used caused a significant biological reaction, such as an increase in phosphorylated Akt. It is therefore possible that our dosing strategy was insufficient to cause a cellular response. In a previous mouse model the total amount of TB4 recovered from the heart was very small compared to the dose originally administered (Mora et al., 1997). In our opinion, optimal cardioprotective tissue and blood concentrations should be determined in order to decide whether intravenous administration in its current form and if dosages used in studies on rodents are adequate also in larger mammals.

We failed to show any significant cardioprotective effect of TB4 in this animal model for global myocardial I-RI. Although previous data is encouraging, to date only a few studies have been performed using large animal models. Overexpression of TB4 by viral vector gene transfer proved beneficial in a pig model for heterotopic heart transplantation (Postrach et al., 2014). In a balloon-occlusion model using pigs there was a decrease in infarct size, inflammation and apoptosis after 1 h of unprotected local ischemia and 24 h of reperfusion when TB4 was infused at the site of injury at the end of the ischemic period (Hinkel et al., 2008). In our study, the setting was different and mimics the conditions during normal CPB assisted open-heart surgery where cardioplegia protects the heart from ischemic insult, placing more emphasis on cardiac injury during reperfusion. The model is also different in that it does not cause myocardial infarction but global and diffuse heart injury.

Macroscopic post-mortem analysis of the two animals that died did not reveal any obvious cause of death. Bradycardia is a known problem after CPB and could be related to damage to the sinoatrial node or the conduction system. The pathogenesis of circulatory failure is usually multifactorial and was here probably associated with CPB induced systemic inflammation and myocardial damage. This study was, however, not designed to investigate survival so we cannot conclude whether TB4 treatment influenced mortality. Our results show that there was substantial cardiomyocyte injury following the procedure as demonstrated by the increase in post-operative cTnT levels and the rate of apoptosis. TB4 did, however, not influence the rate of myocardial cell death. The lack of Gadolinium-contrast uptake on late-enhancement scans showed that there were no larger areas of non-viable myocardium, indicating that the cell necrosis caused in this model is diffuse and not detectible by conventional MRI imaging. Reduction in cardiac function after I-R injury is either permanent or transient. Necrosis and apoptosis leads to a reduced number of working cardiomyocytes, hence impairing cardiac work more or less permanently. Cardiac stunning is a phenomenon seen after different types of cardiac insult and is recognized as a transient decrease in cardiac contractility while blood-flow of the myocardium remains unchanged (Kloner and Jennings, 2001a,b). In this study, left ventricle EF was reduced in both study groups with a slight, although insignificant benefit for TB4 treated animals. There was also a trend for larger diastolic and systolic dimensions of the left ventricle in control animals. We did not record baseline values with either PET or cMRI. Based on previous experience with pigs of similar size and origin we know that baseline LVEF is 64–74% and that global I-RI causes an 8–15% reduction in left ventricle EF in early reperfusion (Vähäsilta et al., 2001; Malmberg et al., 2006, 2011). The complete duration of this stunning effect is unknown but is thought to begin resolving within the first 2 days after I-RI. Impaired cardiac contractility in the immediate postoperative period can; however, be troublesome and cause substantial morbidity to patients (Kloner and Jennings, 2001a,b)

A no-reflow phenomenon has been associated with cardiac I-RI and we wanted to determine global myocardial perfusion and possible effects by TB4 treatment in the subacute phase of I-RI. Coronary flow reserve was low in both study groups. This can be related to increased basal blood flow or inability of the coronaries to respond to adenosine stimulation. At the time of imaging the animals’ heart rates were high and blood pressure values quite low. Adenosine stress might have interfered with the autoregulation of the coronary circulation leading to inability to increase MBF despite maximal vasodilatation. Some correlation between cTnT levels in plasma and MBF during rest was observed which could indicate an association between the extent of cell injury and hyperemia. In preclinical models using unprotected local myocardial I-RI injury, blood flow is decreased early after reperfusion and remains low during the first 24 h (McFalls et al., 1995). In studies with protected global I-RI, MBF has been showed to increase immediately after ischemia, then decrease within the first hours of reperfusion and later rise to normal or even higher levels. In some situations this hyperdynamic blood flow is still observed several days after surgery, the clinical significance of this is, however, unclear (Vähäsilta et al., 2001; Aburawi et al., 2009). Measuring MBF at an earlier time point might have showed signs of malperfusion and could have highlighted some differences between the groups.

The prolonged time on mechanical ventilation and the use of inotropic agents in the postoperative period are factors that could influence several outcome measurements in this study. The use of these drugs was often necessary in order to successfully wean the animals from CPB as it also is in clinical situations. Keeping the animals sedated for the whole follow-up period was considered to be appropriate as recovery from anesthesia and re-intubation for imaging procedures would have caused additional stress to the animals increasing the possibility for complications such as hypoxemia and arrhythmias.

We here partly reached our conclusions regarding functional outcome based on historical data from similar experiments performed by our group. It is possible that the current model didn’t cause a sufficient therapeutic window for TB4 treatment as no baseline imaging procedures were performed or sham-operated animals used. *Post hoc* sample size calculations for a power of 0.8 with the EF values obtained from the study (56.7 vs. 61.9, *SD* 7.6) gave a sample size of 34/group. The *post hoc* calculated power in our study was 0.12. Sample size based on EF was originally calculated with a 10% (EF 55% versus 65%) improvement and a variance of 6 *SD*. The observed difference was therefore 4.8% lower and the variance 1.6 *SD* higher than calculated. Since this is the first study of its kind, hypothetical effect sizes were generated based on results from similar IR-I studies with pigs and animal models with myocardial infarction and TB4 treatment. A 5% difference in EF during the post-operative period is in our opinion not clinically significant compared to 10% with the model used. For a *post hoc* power of 0.8, an increase in EF of 15% would have been necessary with the current sample size and observed variance. Based on the small difference in EF between the groups, we consider this to be a true negative (neutral) observation. Increasing sample size would probably not have influenced effects size although it would have provided more power.

## Conclusion

With regard to the limiting factors addressed above, we conclude that TB4 treatment was not beneficial in this model for global myocardial I-RI assessed by several different markers for cardiac damage. Although promising in studies conducted on rodents, the cardioprotective potential of TB4 has not been well established in large animal models or clinical trials. Previous studies performed on pigs have all relied on local myocardial delivery or cardiac overexpression of the peptide. Further studies are therefore needed to clarify whether systemic administration also is an option in other species and also to determine sufficient dosing strategies. The peptides cellular functions have been investigated quite extensively but more clinically oriented studies are needed in order to translate its therapeutic potential from bench to bedside. This study offers novel information on some potentially significant biological actions of the peptide related to blood coagulation and vasomotor function and can serve as a reference in planning future studies related to cardioprotection and TB4.

## Author Contributions

The study was planned by CS, TS, and JKo. Animal experiments were performed by CS, MT, RK, MM, TV, MS, V-VH, JJ, and TS. Imaging and biochemical analyzes were performed by CS, MT, SR, MH, PT, T-PA, AS, JKn, and JKo. All authors approved the manuscript.

## Conflict of Interest Statement

The authors would like to state that Thymosin beta 4 was provided by RegeneRx Biopharmaceuticals Inc, Maryland, USA.

## Footnotes

1 http://www.turkupetcentre.fi/carimas
